# Supplementary material for: Factor Structure of the KABC-II at Ages 5 and 6: Is It Valid in a Clinical Sample?
Source: Children (Basel). 2022 Apr 30;9(5):645. doi: 10.3390/children9050645 (PMC9139365; doi:10.3390/children9050645)
Supplement: Supplementary file 1 [file children-09-00645-s001.zip › children-1668835-supplementary.pdf]

children

MDPI

*Article*

# Factor structure of the KABC-II at Ages 5 and 6: Is it Valid in a Clinical Sample?

**Gerolf Renner [1,*], Dieter Irblich [2] and Anne Schroeder [3]**

[1] Faculty of Special Education, Ludwigsburg University of Education, 71634 Ludwigsburg, Germany
[2] Formerly Social Pediatric Center, 55469 Simmern, Germany; d.irblich@t-online.de
[3] Werner Otto Institute, 22337 Hamburg, Germany; aschroeder@werner-otto-institut.de
* Correspondence: renner@ph-ludwigsburg.de

# Supplementary Materials

**Table S1**. Intercorrelations of KABC-II core subtests

**Table S2.** Loadings of subtests on the general factor in unidimensional measurement models

**Table S3.** Loadings of CHC model core subtests for ages 5 and 6, and variance explained by g and broad abilities

**Figure S1.** Second-order and bifactor models with standardized loading coefficients for the KABC-II Luria core subtest configuration at age 5.

**Figure S2**. Second-order and bifactor models with standardized loading coefficients for the KABC-II Luria core subtest configuration at age 6.

**Table S1**. Intercorrelations of KABC-II core subtests

| | **Num Rec** | **Word Order** | **Rover** | **Cnpt Think** | **Tri** | **Patt Reas** | **Atl** | **Rebus** | **Rid** | **Expr Voc** |
|---|---|---|---|---|---|---|---|---|---|---|
| Number Recall | — | 0.62 | — | 0.39 | 0.41 | 0.41 | .044 | 0.38 | 0.50 | 0.41 |
| Word Order | 0.67 | — | — | 0.45 | 0.48 | 0.52 | 0.38 | 0.36 | 0.49 | 0.44 |
| Rover | 0.29 | 0.41 | — | — | — | — | — | — | — | — |
| Conceptual Thinking | 0.40 | 0.48 | 0.55 | — | 0.49 | 0.53 | 0.29 | 0.33 | 0.51 | 0.43 |
| Triangles | 0.37 | 0.45 | 0.55 | 0.55 | — | 0.41 | 0.35 | 0.35 | 0.39 | 0.31 |
| Pattern Reasoning | 0.43 | 0.49 | 0.62 | 0.63 | 0.62 | — | 0.30 | 0.46 | 0.47 | 0.40 |
| Atlantis | 0.33 | 0.38 | 0.32 | 0.36 | 0.34 | 0.43 | — | 0.36 | 0.41 | 0.37 |
| Rebus | 0.36 | 0.40 | 0.26 | 0.43 | 0.37 | 0.46 | 0.47 | — | 0.42 | 0.37 |
| Riddles | 0.44 | 0.51 | 0.47 | 0.51 | 0.43 | 0.47 | 0.43 | 0.34 | — | 0.77 |
| Expressive Vocabulary | 0.37 | 0.40 | 0.29 | 0.37 | 0.34 | 0.32 | 0.41 | 0.31 | 0.75 | — |

*Note.* Correlations for ages 5 (above the diagonal) and 6 (below the diagonal). All correlations are significant with $p < 0.001$.

**Table S2**. Loadings of subtests on the general factor in unidimensional measurement models

| | Age 5 | | Age 6 | |
|---|---|---|---|---|
| | **CHC model** | **Luria model** | **CHC model** | **Luria model** |
| Riddles | 0.79 | --- | 0.72 | --- |
| Expressive Vocabulary | 0.71 | --- | 0.58 | --- |
| Rover | --- | --- | 0.67 | 0.69 |
| Conceptual Thinking | 0.65 | 0.64 | 0.74 | 0.75 |
| Triangles | 0.59 | 0.64 | 0.70 | 0.72 |
| Pattern Reasoning | 0.66 | 0.68 | 0.77 | 0.83 |
| Atlantis | 0.54 | 0.52 | 0.56 | 0.53 |
| Rebus | 0.56 | 0.55 | 0.56 | 0.56 |
| Number Recall | 0.67 | 0.70 | 0.60 | 0.57 |
| Word Order | 0.71 | 0.76 | 0.69 | 0.66 |

*Note.* Values are based on models 1b.

**Table S3**. Loadings of CHC model core subtests for ages 5 and 6, and variance explained by g and broad abilities

| Subtest | 5-year-olds | | | | | 6-year-olds | | | | |
|---|---|---|---|---|---|---|---|---|---|---|
| | Loadings | | Sources of variance | | | Loadings | | Sources of variance | | |
| | g | broad | g | broad | unique | g | broad | g | broad | unique |
| Riddles | 0.73 | 0.95 | 0.53 | 0.37 | 0.10 | 0.71 | 0.98 | 0.50 | 0.45 | 0.05 |
| Expressive Vocabulary | 0.62 | 0.81 | 0.39 | 0.27 | 0.35 | 0.56 | 0.77 | 0.31 | 0.28 | 0.41 |
| Rover | | | | | | 0.62 | 0.73 | 0.38 | 0.14 | 0.47 |
| Conceptual Thinking | 0.65 | 0.72 | 0.43 | 0.09 | 0.49 | 0.65 | 0.76 | 0.42 | 0.16 | 0.41 |
| Triangles | 0.58 | 0.64 | 0.34 | 0.07 | 0.59 | 0.63 | 0.73 | 0.39 | 0.15 | 0.46 |
| Pattern Reasoning | 0.66 | 0.72 | 0.43 | 0.09 | 0.48 | 0.71 | 0.84 | 0.51 | 0.19 | 0.30 |
| Atlantis | 0.54 | 0.58 | 0.29 | 0.04 | 0.66 | 0.57 | 0.69 | 0.33 | 0.15 | 0.53 |
| Rebus | 0.57 | 0.61 | 0.33 | 0.05 | 0.62 | 0.56 | 0.67 | 0.31 | 0.14 | 0.55 |
| Number Recall | 0.67 | 0.77 | 0.45 | 0.14 | 0.41 | 0.60 | 0.76 | 0.36 | 0.21 | 0.43 |
| Word Order | 0.71 | 0.81 | 0.50 | 0.16 | 0.34 | 0.70 | 0.89 | 0.49 | 0.29 | 0.22 |

*Note.* Values are based on model 2b.

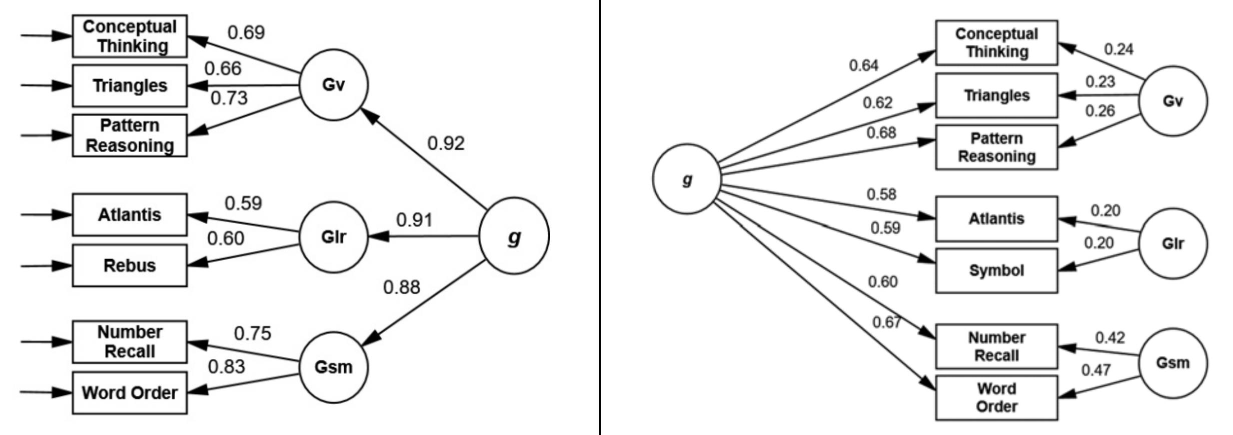


$\chi^2$ = 27.90, df = 15, *p* = .022, CFI = .976, RMSEA = .059, SRMR = .036
(a)

$\chi^2$ = 36.96, df = 24, *p* = .119, CFI = .980, RMSEA = .052, SRMR = .050
(b)

**Figure S1**. Second-order model 2a (a) and bifactor model 3a (b) with standardized loading coefficients for the KABC-II Luria core subtest configuration at age 5.

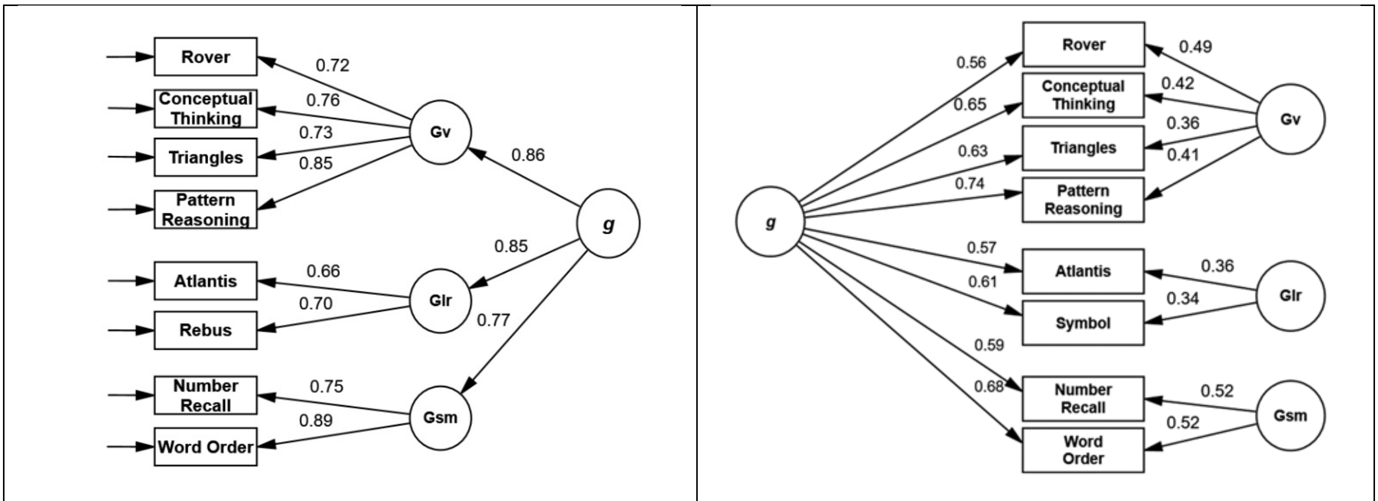


$\chi^2$ = 14.03, df = 17, $p$ = .764, CFI = 1.000, RMSEA = .000, SRMR = .025
(a)

$\chi^2$ = 10.98, df = 17, $p$ = .862, CFI = 1.000, RMSEA = .000, SRMR = .020
(b)

**Figure S2**. Second-order model 2b (a) and bifactor model 3b (b) with standardized loading coefficients for the KABC-II Luria core subtest configuration at age 6.
